# Pitfalls and limitations in translation from biomarker discovery to clinical utility in predictive and personalised medicine

**DOI:** 10.1186/1878-5085-4-7

**Published:** 2013-02-25

**Authors:** Elisabeth Drucker, Kurt Krapfenbauer

**Affiliations:** 1 Department of Molecular Biotechnology, University of Applied Science Vienna, Helmut-Qualtinger-Gasse 2, Vienna A-1030, Austria; 2 Department of Otorhinolaryngology, Medical University of Vienna, Head and Neck Surgery, Waehringer Guertel 18-20, Vienna A-1090, Austria

**Keywords:** Predictive medicine, Targeted prevention, Validation strategies, Regulatory overview, Biomarker perspectives, Tailored therapy

## Abstract

Since the emergence of the so-called *omics* technology, thousands of putative biomarkers have been identified and published, which have dramatically increased the opportunities for developing more effective therapeutics. These opportunities can have profound benefits for patients and for the economics of healthcare. However, the transfer of biomarkers from discovery to clinical practice is still a process filled with lots of pitfalls and limitations, mostly limited by structural and scientific factors. To become a clinically approved test, a potential biomarker should be confirmed and validated using hundreds of specimens and should be reproducible, specific and sensitive. Besides the lack of quality in biomarker validation, a number of other key issues can be identified and should be addressed. Therefore, the aim of this article is to discuss a series of interpretative and practical issues that need to be understood and resolved before potential biomarkers become a clinically approved test or are already on the diagnostic market. Some of these issues are shortly discussed here.

## Review

### Introduction

The strengthening of the robustness of discovery technologies, particularly in genomics, proteomics and metabolomics, has been followed by intense discussions on establishing well-defined evaluation procedures for the identified biomarker to ultimately allow the clinical validation and then the clinical use of some of these biomarkers.

The ability of biomarkers to improve treatment and reduce healthcare costs is potentially greater than in any other area of current medical research. For example, the American Society of Clinical Oncology estimates that routinely testing people with colon cancer for mutations in the *K-RAS* oncogene would save at least US $600 million a year [1]. On the other side, thousand of papers in the course of biomarker discovery projects have been written, but only few clinically useful biomarkers have been successful validated for routine clinical practice [2]. The following are the major pitfalls in the translation from biomarker discovery to clinical utility:

1. Lack of making different selections before initiating the discovery phase.

2. Lack in biomarker characterisation/validation strategies.

3. Robustness of analysis techniques used in clinical trials.

Each of these details is rarely documented and can dramatically affect the predictive outcome of biomarker results. However, the selection of useful biomarkers must be carefully assessed and depends on different important parameters, such as on sensitivity (it should correctly identify a high proportion of true positive rate), specificity (it should correctly identify a high proportion of true negative rate), predictive value etc. Unfortunately, biomarkers with ideal specificity and sensitivity are difficult to find. One potential solution is to use the combinatorial power of different biomarkers, each of which alone may not offer satisfaction in specificity or sensitivity. Besides traditional immunoassays such as ELISA, recent technological advances in protein chip and multiplex technology offer a great opportunity for the simultaneous analysis of a large number of different biomarkers in a single experiment, which has expanded at a rapid rate in the last decade. However, although many significant results have been derived, one additional limitation has been the lack of characterisation and validation of such technologies. Besides technical characterisation, it also needs quality requirements for correct characterisation of the predictive value of biomarkers. In order to overcome these limitations, some authorities (e.g. Food and Drug Administration (FDA), European Medicines Agency (EMA), European Association for Predictive, Preventive and Personalised Medicine (EPMA), National Institute of Health (NIH)) already set up recommendations, short proposals and minimum information about a variety of bio-analytical experiments that describe the minimal requirements to ensure that the technical performance as well as the predicted value of biomarkers are correct. For example, EPMA tries to outline a number of key issues in research, development and clinical trial studies, including those associated with biomarker characterisation, experimental design, analytical validation strategies, analytical completeness and data managements [3]. Actual paper follows recommendation presented in the ‘EPMA White Paper’ [4]. Current recommendations should serve a set of criteria, which will help to carry on to a high-quality data project. Improvements in the quality outcomes are important because without requirements in the improved selection of biomarkers, correct performance of standardisation and validation, the interpretation of the results as well as the direct comparisons of the predictive value of biomarkers between different research labs or clinical trial studies is not possible. Besides the lack of quality in biomarker selection, a number of other key issues can be identified, which should be addressed in the course of this article. Therefore, the aim of this article is to review and discuss a series of interpretative and practical issues that need to be understood and resolved before potential biomarkers go into the market and become feasible diagnostic tools. The content and structure of the necessary information, as well as potential pitfalls and limitations of biomarker research and validation, are discussed briefly in the next subsection.

### Short overview of different kinds of biomarkers

One of the goals of personalising medicine is to use the growing understanding of biology so that patients receive the right drug for their disease, at the right dose and the right time. Although the definitions of personalising vary, they all include the use of different biomarkers driven by a decision-making process in which a diagnostic test is pivotal. Biomarkers include gene expression products, metabolites, polysaccharides and other molecules such as circulating nucleic acids in plasma and serum, single-nucleotide polymorphism and gene variants. Ideal biomarkers for use in diagnostics and prognostics, and for drug development and targeting, are highly specific and sensitive [5]. Biomarkers can also be categorised as pharmacodynamic, prognostic or predictive [6]:

1. Pharmacodynamic biomarkers indicate the outcome of the interaction between a drug and a target, including both therapeutic and adverse effects [7].

2. Prognostic biomarkers were originally defined as markers that indicate the likely course of a disease in a person who is not treated [8]; they can also be defined as markers that suggest the likely outcome of a disease irrespective of treatment [9,10].

3. Predictive biomarkers suggest the population of patients who are likely to respond to a particular treatment [8,9].

**Figure 1:**
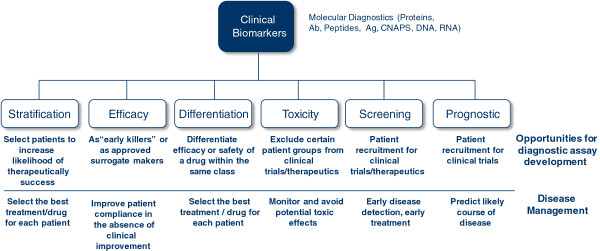
Clinical biomarkers: categories/types.

Predictive biomarkers help to assess the most likely response to a particular treatment type, while prognostic markers show the progression of disease with or without treatment. In contrast, drug-related biomarkers indicate whether a drug will be effective in a specific patient and how the patient’s body will process it. Figure 1 gives an overview of different biomarker categories and types.

In Figure 1, the clinical biomarkers for diagnostics determine whether a patient is suitable for treatment with a particular drug (by stratification markers), determine the most effective dose for the patient (by efficacy markers), determine the underlying susceptibility of a patient for a particular side effect or group of side effects (by toxicity markers) or evaluate the course and effectiveness end point of a therapy (by surrogate endpoint markers).

Biomarkers can also be used as surrogate end points (end points that substitute for a clinical outcome such as how a patient feels or functions, or how many patients survive) [9,11,12]. Another way of classifying biomarkers is by their role in drug development. Pharmacokinetic or pharmacodynamic biomarkers are involved in early preclinical to phase I studies, and clinical (prognostic, predictive and surrogate) biomarkers play a role in phase II and III trials [10].

The Biomarkers and Surrogate End Point Working Group [13] has defined a classification system that can be used for biomarkers [14]:

1. Type 0 consists of disease natural history biomarkers that correlate with clinical indices;

2. Type I tracks the effects of intervention associated with drug mechanism of action;

3. Type II consists of surrogate end points that predict clinical benefit.

Measurement of different markers (RNA, DNA and/or proteins) needs different diagnostic assays; therefore, different qualification and validation strategies are required.

Pharmaceutical companies are increasingly looking to develop a drug and diagnostic test simultaneously, in a process referred to as drug-diagnostic-co-development so-called companion diagnostic (CDx), to better define the appropriate patient population for treatment. CDx are increasingly important tools in drug development because they lead to the following:

1. Reduced costs through pre-selected (smaller) patient population;

2. Improved chances of approval;

3. Significantly increased market uptake;

4. Added value for core business (late phase);

5. Regulatory trend to have CDx mandatory.

The first drug introduced using the personalised medicine paradigm—Herceptin (Trastuzumab; Roche/Genentech, South San Francisco, CA, USA)—has now been on the market for more than a decade. However, the number of drugs marketed alongside CDx remains small (see Table 1).

**Table 1:** Overview of already approved CDx on the markets

**Biomarker**	**Related drug**	**Company**	**Indication**	**Test**
Her-2/neu	Herceptin	Genentech/Roche	Breast cancer	PathVysion®FISH
Kit (CD117)	Gleevec/Glivec	Novartis	Gastrointestinal	c-Kit pharmDx
EGFR	Erbitux/Tarceva	Bristols-Myers/Genentech	Colorectal/NSCLC	EGFR pharmDx kit
CD20	Rituxan/Bexxar	Genentech/Glaxo	NHL	Flow cytometry
CD25	Ontak/Onzar	Eli Lilly	Lymphoma	Flow cytometry
CD33	Mylotarg	Wyeth	Leukaemia, CML	Flow cytometry
Estrogen receptor	Nolvadex	AstraZeneca	Breast cancer	Hormone receptor assay
HLA A2/HLA C3	Melacine	GlaxoSmithKline	Melanoma	Serology, DNA-based
Philadelphia chromosome	Roferon-A/Gleevec/Glivec	Roche/Novartis	Leukaemia, CML	BCR-ABL chromosome translocation test
T(15;17) translocation	Trisenox	Cephalon	Leukaemia, CML	Fluorescence *in situ* hybridisation (FISH)
PML/RAR-α gene expression	Vesanoid	Roche	Leukaemia, CML	

EGFR, epidermal growth factor receptor; CML, chronic myelogenous leukaemia; NSCLC non-small-cell lung carcinoma; NHL, non-Hodgkin lymphoma.

Regulatory hurdles have been cited as other main reasons for the slow growth in this area. The differences between the regulatory process in the European Union (EU) and USA and the complexities of the regulatory processes in both regions cause other huge problems for companies. These difficulties affect the preparation of dossiers and their timing and are amplified when considering a CDx project, particularly where more than one company (e.g. pharmaceutical and diagnostic companies) is involved.

Advances in the science underlying drug development have made the discovery of novel biomarkers a real possibility, whilst still challenging, and the use of biomarkers to drive drug development programmes has been increasing steadily over the past decade. Whilst the majority of these biomarkers will not be translated into CDx tests, the growth of biomarker use indicates that the future of the industry will lie in personalised medicine.

**Figure 2:**
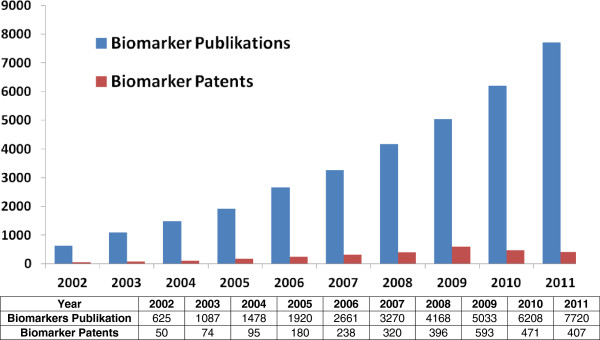
Overview of the relationship between publications and patenting of biomarkers.

As reflected in Figure 2, the search of the scientific literature indicates that many studies report the discovery of different potential biomarkers, but most of them do not meet the criteria of high sensitivity and specificity. The lack of sensitivity and/or specificity leads to a low number of patent application and, in addition to this, to a low number of successful market applications.

If the biomarker used for patient selection is known from the earliest stages of the development process, the process of assay development can begin early, and there will be a selection of diagnostic assay used in clinical trials from an early stage. Biomarkers related to response to therapy are often the result of clinical investigations in patients and may not be available until later in the development programme.

Diagnostic development is undertaken in three stages once a biomarker has been identified. Analytical validation ensures the consistency of the test in being able to measure the specific biomarker. Clinical validity relates to the consistency and accuracy of the test in predicting the clinical target or outcome claimed, and clinical utility relates to the fact that the test should improve the benefit/risk of an associated drug in the selected and non-selected groups. Table 2 describes strategic consideration and implication positions of key stakeholders—regulators, pharma and diagnostics companies, patients, physicians and healthcare providers.

**Table 2:** Strategic considerations and implications of personalised medicine

	
Pharmaceutical companies	• Generate new revenue stream
	• Increased targeted therapies
	• Improve current clinical trials design (kick-off candidates at phase III)
	• Differentiate CM product offerings
	• Shorten clinical trials
	• Improve go-no-go decisions in clinical trials and make it earlier
Diagnostic companies	• New pivotal in the personalised medicine
	• Need to establish relationships with pharmaceutical companies
Payers/health ensurers	• Payers ensure payment of personalised medicine
	• Agree on reimbursement
	• Improve the availability of personalised medicine and their respective diagnostic
	• Have control over escalating healthcare costs
Regulatory authorities	• Clinical trials with improved statistical relevance
	• Will aid co-development programmes
	• Enhance the utility of test information on product labelling

Case studies of drugs and their companion diagnostics that have been approved over the last 10 years indicate that the number of co-developed products is small. The majority of diagnostic tests available to drive patient selection for particular drugs have been added years after the drug’s approval. However, experience from the EU and USA also indicates that regulators will not approve targeted drugs in the absence of available, relevant diagnostic tests.

### Key points to be addressed

According to Issaq et al. [5], the failure in finding high-sensitive and high-specific biomarkers may be attributed to the following factors:

1. Small number of samples that are analysed;

2. Lack of information on the history of the samples;

3. Case and control specimens which are not matched with age and sex;

4. Limited metabolomic and proteomic coverage; and

5. The need to follow clear standard operating procedures for sample selection, collection, storage, handling, analysis and data interpretation.

Furthermore, most studies to date used samples with a complex matrix such as serum, plasma, urine or tissue from patients and controls. Another reason for pitfalls in biomarker validation is the usually slow progression of some diseases, requiring high numbers of well-stratified patients who are undergoing long-term treatment when conventional diagnosis and imaging techniques are used. Importantly, there is a lack of sensitive and specific prognostic biomarkers for disease progression or regression that would permit a rapid clinical screening for potential responders and non-responders. Nonetheless, in view of an urgent need for novel therapeutics that have a positive impact on morbidity and mortality of chronic diseases, the field is now moving more quickly towards clinical translation. This development is driven by smart preclinical validation, a better study design and improved surrogate readouts using currently available methodologies and diagnostic techniques. Moreover, upcoming novel biomarkers and diagnostic technologies will soon permit a more accurate and efficient assessment of disease progression and regression.

### Considerations before initiating the biomarker discovery phase

Although some biomarkers have been approved by the FDA as qualitative tests for monitoring specific diseases (e.g. nuclear matrix protein-22 for bladder cancer), unfortunately, the majority of found biomarkers (proteins or metabolites) are not sensitive and/or specific enough to be used for population screening. One of the major reasons that proteomic and metabolomic studies over the past decade have failed to discover molecules to replace existing clinical tests is due to errors in either study design and/or experimental execution. Werner Zolg wrote in a review [15] that, before initiating the discovery phase, the first step in the process chain of creating new diagnostic content is to make critical decisions on the sample selection that will directly impact the outcome of the identification process. The very selection of the discovery samples and their degree of characterisation of the material, down to the standard operation procedures on how the samples were acquired and stored, can be decisive for success or failure. By selecting tissue as the discovery material for biomarker identification, one must inevitably choose between cultured cells or specimen directly obtained from patients. There are advantages/limitations to either option.

### Consideration on the selection and randomisation of patients for biomarker studies: looking for the ‘ideal’ patients

The optimal selection and randomisation of patients is essential and has to be included in each clinical trial, testing the efficacy of drugs and biomarkers. In particular, given the variant course of disease progression even in well-selected patients with a dominant single aetiology, subjects should be well matched according to factors such as the following lifestyle risk factors: (1) alcohol and tobacco consumption, (2) body mass index, (3) physical activity, (4) signs of the metabolic syndrome or (5) use of (over-the-counter) medications. As in other studies, age and sex should be balanced. In addition, stratification of patients as to their genetic risk of developing a specific disease, (e.g. using a score) will be central to obtaining a balanced randomisation of the placebo vs. the treatment group. These facts alone should significantly reduce the number of patients and the duration of the trial needed to demonstrate a significant reduction of disease progression or induction of regression. Histological end points in proof-of-concept trials will still be required by regulatory authorities, apart from long-term hard end points, such as morbidity and mortality in phase III trials. At present, it is not possible to exactly predict the number of patients and the time on treatment that are needed to demonstrate the clinical benefit of a drug agent or biomarker. This is one major reason that companies have been reluctant to enter this difficult field.

### The current state of biomarker discovery

The search of the scientific literature clearly indicates that most published biomarkers are inadequate to replace an existing clinical test or that they are only useful for detecting advanced disease stage, where the survival rate is low. Many molecular or genetic biomarkers have been suggested for the detection of different diseases; however, most of them do not possess the required sensitivity and specificity. Another reason why most proposed metabolomic and proteomic biomarker results that have not progressed from the laboratory to the clinic study is that the majority stopped at the first phase of biomarker discovery. According to other studies [5,16,17], there are five phases that a protein or a metabolite has to go through to become a biomarker. Phase I is preclinical exploratory studies to identify potentially useful markers, phase II is clinical assay development for clinical disease, phase III is retrospective longitudinal repository studies, phase IV is prospective screening studies and phase V is control studies [5].

Listed examples of already approved biomarkers in Table 1 show that there are no 100% sensitive and specific biomarkers for different types of diseases to date. A biomarker with a high sensitivity has a low specificity and vice versa. Unfortunately, biomarkers with ideal specificity and sensitivity are difficult to find. One potential solution is to use the combinatorial power of a number of different biomarkers, each of which alone may not offer satisfactory in specificity. For example, Horstmann et al. [18] studied the effect of using a combination of bladder cancer biomarkers on sensitivity and specificity. Although none of the combinations resulted in 100% sensitivity and specificity, the sensitivity improved from 91% (using two biomarkers) to 98% using a combination of four different biomarkers.

### Pitfalls and limitations

However there exist different reasons why most potential biomarkers failed in achieving adequate sensitivity and specificity and are not accepted as clinical tests. One main reason is that most biomarkers are dealing with detecting diseases at an early stage in humans that have different age, sex and ethnicity. Other important fact is to find a protein or a metabolite at an extremely low concentration level among thousands of other proteins and metabolites. To improve sensitivity and specificity, there are different strategies: potential solutions are listed as follows:

1. Improve the assay (e.g. antibody with a higher specificity and/or in combination with a detection conjugate with a higher sensitivity),

2. Combine several markers,

3. Check for subpopulations and stratify population (e.g. matched by gender, age, pathology).

The current procedure for the search of biomarkers is dealing with potential errors in the study design that can be avoided in future studies.

**Figure 3:**
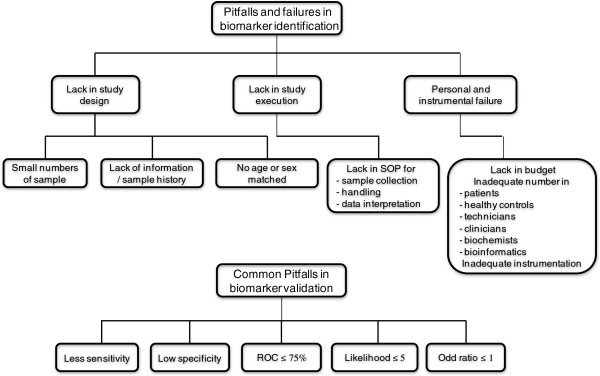
Pitfalls and failures in biomarker identification.

Figure 3 gives an overview about two main reasons why most potential biomarkers failed in achieving adequate sensitivity and specificity and are not accepted as clinical tests. One main reason is pitfalls and limitations in biomarker discovery and second main reason is pitfalls in biomarker validation.

### Age, sex and race

Biomarker studies are normally carried out using body fluids or tissues collected from patients and healthy subjects of different ages, sex and race. Using samples from patients and controls that are of different ages and sexes can dramatically influence the results. In a recent study, Lawton et al. used 269 subjects, 131 males and 138 females, to study the effects of age, sex and race on plasma metabolites. The patients were of Caucasian, African-American and Hispanic descent and ranged in age from 20 to 65 years. The subjects were divided into three different age groups; 20–35, 36–50 and 51–65. Using GC/mass spectrometry (MS) and high-performance liquid chromatography (HPLC)/MS, they reported that ‘more than 300 metabolites were detected of which more than 100 metabolites were associated, with age, many fewer with sex and fewer still with race’ [19].

### Selection of patients and controls

Patients for biomarker studies should be carefully selected by a specialist (e.g. oncologist for cancer studies or a pathologist for tissue samples) to insure the presence or absence of diseases. Unfortunately, predictive curve values of biomarkers with no or less overlapping of diseased vs. non-diseased cohorts are difficult to find. There exist always more or less overlapping areas between healthy and diseased cohort. The overlapping area allows the analyst to calculate the proportion of patients whose diagnosis was correctly predicted by the model (true positives for sick patients and true negatives for healthy patients) or false negative or false positive values [3].

Generally, the number of patients and control subjects in published studies is very small to give an acceptable statistical value. Also, many of the potential proposed markers have not been confirmed or validated in a high-quality manner. Body fluids and tissues are collected from a group of patients of different disease stages, and results are compared with a group of healthy persons. The effect of a disease stage on sensitivity of a single biomarker should be taken into consideration as mentioned previously because sensitivity improves with increase in disease stage. Grossman [20] adequately summarises the importance of consistency through his observation that ‘the contradicting published reports likely [resulted] from studies testing different patient populations, using different methodologies, and applying different [cut-offs] for a positive test’.

### Errors in study execution

Study execution deals with experimental parameters that need to be considered. These parameters include many different variables, such as sample collection, handling and storage, sample comparison, number of samples, sample preparation, methods of analysis and number of replicates.

### Sample collection, handling and storage

Samples are collected from a person who passed a physical exam by a physician who determines that the person of interest has a concrete disease or is healthy. Samples (serum, plasma, urine, saliva, tissue etc.) should be collected in freezer-type tubes, immediately snap frozen and stored in a freezer until time of analysis. It is recommended that, for short-time storage (less than 1–2 weeks), storage condition should be at −20°C, and for long-term storage (more than 2 weeks), storage condition should be at −70°C. At the time of analysis, samples should be thawed at 4°C or on ice and prepared according to the selected method of analysis. The history of the sample is very important and may have been obtained from sample storage banks with proper collection, storage information about the stage of disease, medication, pathology, age, gender and condition of patients. A lack of consistency in sample collection and storage can doom a study before any data are even collected.

### Direct sample comparison

If this option is chosen, the degree of sample characterisation is critical. It is of importance that the specimens used in the diseased cohort are not simply classified as ‘diseased’ (if possible, together with the stage of the disease) but that a detailed histopathological assessment of the distribution of cell types (e.g. tumour cells, necrotic cells, stroma) in the diseased specimens is carried out [5]. This distribution should be as uniform as possible in all samples, and it should represent the correct disease/healthy state. Otherwise, normalising the analytical outcome becomes very difficult.

### Number of samples

The number of samples that have to be placed in the diseased and healthy control groups in order to be compared with a variety of analytical approaches remains a matter of discussion. A minimum of 15 samples in the discovery phase is necessary to get a reasonable representative selection basis for marker candidates. If the number is for practical reasons (resources, cohort and time lines), which is very small (e.g. less than 10 per group), then the observed differences between the two sets of specimens are in danger of being over-interpreted when extrapolated to generalised cohorts. Low sample sizes make the correct identification of those differences increasingly difficult. To overcome these limitations, Zolg [15] recommended running second and third discovery rounds to complement the results of the first round. Ideally, the sample number analysed should not only allow stating the presence or absence of a given protein, but should also give the opportunity to identify trends. Another opportunity is to pool the samples, i.e*.* to physically combine several of the extracts to create fewer samples, to be put through the entire analytical process. Pooling of samples inevitably leads to a loss of information. The distribution of proteins is averaged by the very pooling process with the prospect that individual proteins are pushed below the detection limit by one member of the pooling cohort not expressing the protein in question. At any rate, somewhere in the selection process, the individual spectrum of proteins has to be established. Therefore, the pooling process just shifts the workload to a later point in the process chain, and very good arguments have to be found to deliberately increase the complexity of the data sets by pooling.

### Sample preparation

Preparations of the sample for proteomic and metabolomic analyses prior to analysis are very important and can introduce errors that always will affect the final results [3,5]. The search for biomarkers in biological samples involves different steps depending on the sample type and if it is analysed for metabolites or proteins. Extraction of metabolites from the blood, urine or tissue required multiple purification and extraction procedures using different solvent systems as discussed by Want et al. [21] and Issaq et al. [5]. It is not always possible to extract or to isolate all the metabolites from a sample with a single solvent since metabolites have different chemical and physical properties and are present in a wide dynamic range of concentrations. The search for a protein biomarker involves extraction of the proteins followed by fractionation, purification, specific enrichment and then analysis by different analytical methods (e.g. 2DE-PAGE, immunoassays, Western blot, HPLC/MS/MS). Analysis of the blood as well as the serum is more complicated than that of urine or saliva as it contains fewer proteins, and high-abundant protein must be depleted prior to analysis. Approximately 99% of the protein content of the blood (both serum and plasma) is made up of only about 20 proteins (http://www.plasmaproteome.org) [22]. While depletion of these proteins will allow the detection of low-abundant proteins, it may remove proteins that are bound to these 20 proteins, resulting in a loss of potentially important information [23]. Tissues are homogenised first followed by metabolites, and proteins are extracted and analysed. Incomplete homogenisation can lead to losses that can affect the accuracy of the results. In addition, one cannot ignore human errors in sample collection, storage, weighing, extraction etc.

### Methods of analysis

Choosing the optimal analysis method is critical in biomarker search by proteomics and metabolomics. For example, analysing the plasma proteome involved protein precipitation and solubilisation; therefore, the downstream fractionation method must be either electrophoresis or a liquid-phase method. Unfortunately, studies have shown that the proteome analysis by groups using different methods resulted not only in different numbers of protein identifications, but also in poor overlap between the results [5,23,24]. These results prove that the selected method of analysis is an important parameter.

### Number of replicates

Sample should be analysed in triplicate and report the mean and standard deviation. Unfortunately, most published proteomic and metabolomic studies only analyse each sample once, which does not permit the deviation from the mean (i.e*.* the error in the measurement) to be calculated. Proteomic analysis of a biological sample involves different analytical steps in the course of sample preparation. Each one of these steps can introduce an error. Due to difficulties either in sample preparation, in protein preparation or in assay or protein chip hybridisation, the amount of replicas varied from zero to six. Thus, implicating different optimal statistical tests were necessary for the various settings.

### Consideration on the improvement of current efficacy readouts by development of non-invasive diagnostic tools

Further improvement is desirable to reduce the number of study patients, trial duration, costs and, most importantly, possible risks for individuals. Thus, new innovative diagnostic techniques are needed that allow an exact assessment of the degree of disease and, more importantly, of the dynamic processes underlying the diseases. Such biomarkers and technologies will have to be specific for the targeted structure, i.e*.* the cells or key molecules involved in the development of the disease. Ideally, sensitive and specific markers/imaging methodologies will allow a rapid and mechanism-based screening for and efficacy monitoring of treatments. Additionally, there is a need for universal-standardised reporting methods to aid interpretation and comparison of potential clinical biomarker trails. All current non-invasive methodologies (serum markers, serum marker algorithms, contrast imaging etc.) yield a sufficient to excellent diagnostic accuracy for the detection (or exclusion) of an upcoming or current disease.

### Regulatory outlook and future aspects

The regulatory landscape for biomarker discovery and validation projects (especially for drug-diagnostic co-development = companion diagnostic) is evolving and getting more important to the upcoming clinical trial studies. In the past few years, available data have been reviewed by FDA and EMA, and experience from some exploratory data submission process was used to create a formal biomarker qualification purpose [25].

Both the FDA and EMA have similar biomarker qualification processes in place that enable research institutes and pharmaceutical companies to obtain advice or qualification of the biomarker in question. In both cases, similar guidance concepts were developed that are very clear on the fact that biomarker qualification does not constitute a review of a diagnostic for commercialisation. Nevertheless, for the future, biomarker qualification submissions are strongly recommended by US and EU authorities and will be more and more required for drug/diagnostic co-development projects in both regions [25]. Further guidance on clinical trial enrichment and internal standard operating procedures for cross-labelling efforts are also expected and will improve the penetration of personalised medicine in clinical practice.

The FDA’s first guideline was finalised in 2005, and it is based on the fact that many clinical trial studies were utilising biomarkers but that these data were often exploratory and that their regulatory submission was not required [25]. However, the US regulatory agency regarded the submission of these data as beneficial for both the industry and the FDA to ensure that regulatory scientists are familiar with and are prepared to evaluate future submissions. This data mainly includes pharmacogenomic information, and the programme is referred to as a voluntary exploratory data submission (VXDS). The success of this VXDS programme has led to the development of a number of new (draft) guidance documents including those related to the biomarker qualification process and to clinical pharmacogenomics in the early phases of drug development. Further guidance on clinical trial enrichment and internal standard operating procedures for cross-labelling efforts within FDA offices is also expected and is continuously under discussion.

Since the FDA’s initial publication, the International Committee on Harmonisation (ICH) has published a guidance relating to pharmacogenomic data (ICH E15) that defines pharmacogenomics, pharmacogenetics, genomic biomarkers, and relevant sample and data coding. Standardised terminology is presented for incorporation in future regulatory documents related to pharmacogenetics and pharmacogenomics. Further ICH guidance, topic E16, on the information required for biomarker qualification was published in 2010. In addition, the FDA has established processes for working jointly with EMA on the review of exploratory information. A review of their experience and the impact of the guidance were published in 2010 [16].

## Conclusions

While application of potential biomarkers in preclinical development is far advanced, only a handful have passed clinical trials (see Table 1) and are already commercially successful on the market (see Table 3). Reasons for the pitfalls are manifold, including difficult validation strategies and the usually slow disease progression, requiring high numbers of well-stratified patients undergoing long-term treatment when conventional diagnostic parameters or related end points are used. Importantly, there is a notorious lack of sensitive and specific surrogate biomarkers for disease progression or regression that would permit a rapid clinical screening for potential drug candidates. Nonetheless, in view of an urgent need for new drugs that positively impact morbidity and mortality of different diseases, the biomarker field is now moving more quickly towards clinical translation. This development is driven by thoughtful preclinical validation, a better study design and improved surrogate readouts using currently available methodologies. Moreover, upcoming novel biomarkers and imaging technologies will soon permit a more exact and efficient assessment of disease diagnosis, disease progression and disease regression as already published in other works [26,27].

**Table 3:** Molecular diagnostic players with approved tests

**Manufacturer**	**Headquarter**	**Number of tests**	**Global sales IVD**
Roche Molecular Diagnostics	Switzerland	24	20%
Gen-Probe	CA, USA	18	
Cepheid	CA, USA	13	
Becton, Dickinson and Company	NJ, USA	11	
AdvanDx	MA, USA	10	
Abbott Molecular	IL, USA	8	15%
Hologic	MA, US	7	
Nanosphere	IL, USA	7	
Qiagen	Germany	7	
Idaho Technology	UT, USA	5	
AutoGenomics	CA, USA	4	
bioMerieux	France	4	
Luminex Molecular Diagnostics	TX, USA	4	
Siemens Healthcare Diagnostics	IL, USA	3	15%^a^
*Others*		*28*	
*Total*		*153*	

^a^Mainly imaging technology. After Datamonitor; adapted from the Association of Molecular Pathology.

## Competing interests

The authors declared that they have no competing interests.

## Authors’ contributions

ED helped carry out the economic studies and drafted the manuscript. KK initiated the studies. Both authors read and approved the final manuscript.

## Authors’ information

ED is a student at the University of Applied Science in Vienna and will graduate with a master’s degree in Molecular Biotechnology soon. She specialises in biopharmaceutical technologies for biopharmaceutical production, drug delivery and bioanalytical chemistry. She is a freelance writer with experience in writing for the pharmaceutical industry. KK has been involved in pharmaceutical research for over 12 years as a scientist and project leader within different leading pharmaceutical companies.

## References

[B1] Javle M Hsueh CT Recent advances in gastrointestinal oncology—updates and insights from the 2009 annual meeting of the American society of clinical oncology J Hematol Oncol 2010 2009 23 3 11 23 10.1186/1756-8722-3-11 PMC2856525 20331897

[B2] Poste G Bring on the biomarkers Nature 2011 469 156 157 10.1038/469156a 21228852

[B3] Waerner T Urthaler J Krapfenbauer K The role of laboratory medicine in healthcare: quality requirements of immunoassays, standardisation and data management in prospective medicine EPMA J 2010 1 619 626 10.1007/s13167-010-0053-y 23199116 PMC3405356

[B4] Golubnitschaja O Costigliola V EPMA General reports & recommendations in predictive, preventive and personalised medicine 2012: white paper of the European Association for Predictive, Preventive and Personalised Medicine EPMA J 2012 3 14 10.1186/1878-5085-3-14 23116135 PMC3485619

[B5] Issaq HJ Waybright TJ Veenstra TD Cancer biomarker discovery: opportunities and pitfalls in analytical methods Electrophoresis 2011 32 9 967 975 10.1002/elps.201000588 21449066

[B6] Amur S Frueh FW Lesko LJ Huang SM Integration and use of biomarkers in drug development, regulation and clinical practice: a US regulatory perspective Biomark Med 2008 2 3 305 311 10.2217/17520363.2.3.305 20477416

[B7] Feuerstein GZ Dormer C Jr Rufollo RR Stiles G Walsh FS Rutkowski JL Translational medicine perspectives of biomarkers in drug discovery and development. Part I. Target selection and validation - biomarkers take center stage Int Drug Discovery 2007 2 5 36 43

[B8] Bruenner N What is the difference between “predictive and prognostic biomarkers”? Can you give some examples? Connection 2009 13 18

[B9] Buyse M Sargent DJ Grothey A Matheson A de Gramont A Biomarkers and surrogate end points - the challenge of statistical validation Nat Rev Clin Oncol 2007 7 6 309 317 20368727 10.1038/nrclinonc.2010.43

[B10] Buyse M Michiels S Sargent DJ Grothey A Matheson A de Gramont A Integrating biomarkers in clinical trials Expert Rev Mol Diagn 2011 11 2 171 182 10.1586/erm.10.120 21405968

[B11] Rosenkranz B Biomarkers and surrogate end points in clinical drug development Appl Clin Trials 2003 5 2 30 40

[B12] Lassere MN Johnson KR Boers M Definitions and validation criteria for biomarkers and surrogate end points: development and testing of a quantitative hierarchical level of evidence schema J Rheumatol 2007 34 3 607 615 17343307

[B13] Atkinson AJ Colburn WA DeGruttola VG Biomarkers and surrogate end points: preferred definitions and conceptual framework Clin Pharmacol Ther 2001 69 3 89 95 11240971 10.1067/mcp.2001.113989

[B14] Frank R Hargreaves R Clinical biomarkers in drug discovery and development Nat Rev Drug Discov 2003 2 7 566 580 10.1038/nrd1130 12838269

[B15] Zolg W The proteomic search for diagnostic biomarkers: molecular & cellular Proteomics 2006 5 10 1720–1726 10.1074/mcp.R600001-MCP200 16546995

[B16] Goodsaid FM Mendrick DL Translational medicine and the value of biomarker qualification Sci Transl Med 2010 2 47 47 10.1126/scitranslmed.3001040 20811041

[B17] Pepe MS Etzioni R Feng Z Potter JD Thompson ML Thornquist M Winget M Yasui Y Phases of biomarker development for early detection of cancer J Natl Cancer Inst 2001 93 1054 1061 10.1093/jnci/93.14.1054 11459866

[B18] Horstmann M Patschan O Hennenlotter J Senger E Feil G Stenzl A Combinations of urine-based tumour markers in bladder cancer surveillance Scand J Urol Nephrol 2009 43 461 466 10.3109/00365590903296837 19903092

[B19] Lawton KA Berger A Mitchell M Milgram KE Evans AM Guo L Hanson RW Kalhan SC Ryals JA Milburn MV Analysis of the adult human plasma metabolome Pharmacogenomics 2008 9 383 397 10.2217/14622416.9.4.383 18384253

[B20] Grossman HB Are biomarkers for bladder cancer beneficial? J Urol 2010 183 11 12 10.1016/j.juro.2009.10.052 19913828

[B21] Want EJ O’Maille G Smith CA Brandon TR Uritboonthai W Qin C Trauger SA Siuzdak G Solvent-dependent metabolite distribution, clustering, and protein extraction for serum profiling with mass spectrometry Anal Chem 2006 78 743 752 10.1021/ac051312t 16448047

[B22] Xiao Z Conrads TP Lucas DA Janini GM Schaefer CF Buetow KH Issaq HJ Veenstra TD Direct ampholyte-free liquid-phase isoelectric peptide focusing: application to the human serum proteome Electrophoresis 2004 25 128 133 10.1002/elps.200305700 14730577

[B23] Anderson NL Polanski M Pieper R Gatlin T Tirumalai RS Conrads TP Veenstra TD Adkins JN Pounds JG Fagan R Lobley A The human plasma proteome: a nonredundant list developed by combination of four separate sources Mol Cell Proteomics 2004 3 311 326 10.1074/mcp.M300127-MCP200 14718574

[B24] Buscher JM Czernik D Ewald JC Sauer U Zamboni N Cross-platform comparison of methods for quantitative metabolomics of primary metabolism Anal Chem 2009 81 2135 2143 10.1021/ac8022857 19236023

[B25] Sleigh S Barton C Advances in Drug-Diagnostic Co-Development 2011 London: Scrip Business Insights

[B26] Ausweger C Burgschwaiger E Kugler A Schmidbauer R Steinek I Todorov Y Thurnher D Krapfenbauer K Economic concerns about global healthcare in lung, head and neck cancer: meeting the economic challenge of predictive, preventive and personalized medicine EPMA J 2010 1 4 627 631 10.1007/s13167-010-0054-x 23199117 PMC3405345

[B27] Koehn J Krapfenbauer K Advanced proteomics procedure as a detection tool for predictive screening in type 2 pre-diabetes EPMA J 2010 1 1 19 31 10.1007/s13167-010-0005-6 23199038 PMC3405302

